# Clinical, Radiological, and Immunohistological Distinctions Between Limbic‐Predominant and Typical Alzheimer's Disease: A Systematic Review

**DOI:** 10.1002/brb3.71586

**Published:** 2026-07-09

**Authors:** Guilherme Linha Secco, Kashif Qureshi, Murtaja Satea Shafeea, Pedro Henrique Karpinski, Kim Wouters, Mateus Cordeiro Merlim da Silva, Sravan Reddy Ganta, Enzo von Quednow, Edgar Daniel Guzmán‐Ríos, Khalil St Brice, Mahi Dsouza, Ailton Fernandes, Noor Najah Noori, Bipin Chaurasia, Kivanc Yangi

**Affiliations:** ^1^ State University of Ponta Grossa Ponta Grossa Brazil; ^2^ Department of Neurosurgery Yale School of Medicine New Haven Connecticut USA; ^3^ Department of Neurosurgery, College of Medicine University of Warith Al‐Anbiyaa Karbala Iraq; ^4^ Open University Department of Psychology Heerle The Netherlands; ^5^ Federal University of Parana Parana Brazil; ^6^ Osmania Medical College Hyderabad India; ^7^ Department of Clinical Neurophysiology Former Resident Albacete University Hospital Complex Albacete Spain; ^8^ Department of Medicine Family Justice Center, SSA Tepic México; ^9^ University of the West Indies St Augustine Trindad; ^10^ Georgia Institute of Technology Atlanta Georgia USA; ^11^ State University of Maringa Maringá Brazil; ^12^ College of Medicine University of Baghdad Baghdad Iraq; ^13^ Department of Neurosurgery Neurosurgery Clinic Birgunj Nepal; ^14^ Department of Neurosurgery Barrow Neurological Institute Phoenix Arizona USA

**Keywords:** hippocampal atrophy, limbic‐predominant Alzheimer's disease, neurofibrillary tangles, TDP‐43 pathology

## Abstract

**Background:**

Alzheimer's disease (AD) is the most common cause of dementia worldwide and one of the leading causes of morbidity and mortality among elderly people. It is characterized by generalized brain atrophy, especially affecting the hippocampus and medial temporal lobe. In this context, new subtypes of AD have been documented, including a limbic‐predominant subtype (LP), and the current literature is insufficient to clarify the similarities and differences between these subtypes and the typical presentation. Recently, new studies have proposed a clinical criterion for LP amnestic syndrome, separating it from AD. Therefore, this study aims to evaluate the clinical, radiological, and immunohistological distinctions between those two presentations.

**Methods:**

This study was conducted in accordance with the PRISMA guidelines. Notable databases were utilized for sources: PubMed, Embase, and Web of Science. Baseline characteristics, clinical, radiological, and immunohistological features, and follow‐up times were recorded. Screening was performed using the Rayyan system, and quality assessment was conducted using appropriate tools.

**Results:**

After reviewing 211 articles, screening yielded 21 articles, totaling 11,315 patients. Among these, 1178 (15.7%) presented with LP and 4159 (36.7%) with AD. A total of 5378 (47.6%) had a different presentation, including hippocampal sparing only and the association of LP and typical AD. The weighted average for education in years was 24.31 for LP patients and 17.15 for typical AD patients. The weighted average for age at onset was 72.33 for typical AD patients and 77.36 for LP patients. For the duration of the disease, the weighted average for typical AD was 8.95, and it was 8.43 for LP. There were no differences in clinical presentation, with cognitive impairment and memory deficits being the most cited manifestations. MRI and FDG‐PET are the most commonly used imaging techniques; in typical AD patients, different levels of hippocampal and medial, lateral parietal, and frontotemporal lobe atrophy are observed. In LP patients, imaging findings revealed lower hippocampal volume and higher metabolic rates than in typical AD patients. MRI R2 relaxometry in LP patients revealed lower R2 relaxation rates in the amygdala, hippocampus, and temporal lobe white matter compared with typical AD patients. Tau‐PET imaging in typical AD patients demonstrated elevated standardized uptake value ratios in the parietal and posterior cingulate cortex. The immunohistological findings revealed a greater hippocampal tau burden than in cortical regions and a greater number of TDP‐43 inclusions in LP patients than in typical AD patients. Typical AD patients had a weighted average of 20.06 and LP patients 17.7.

**Conclusion:**

Our analysis of clinical, radiological, and immunohistological features revealed significant differences between LP and typical AD presentations. However, those findings alone cannot reliably determine accuracy, whether both presentations are stages of the same pathology or different diseases. More studies need to explore this field to further examine this topic.

## Graphical abstract

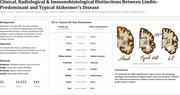

## 1 Introduction

Alzheimer's disease (AD) stands out as the most common form of dementia and one of the leading causes of morbidity and mortality in elderly patients. In the case of AD, brain atrophy occurs in a progressive manner, with considerable shrinkage of the hippocampus and the medial temporal lobe (Knopman et al. 2021). However, AD is not an entirely homogeneous entity. There is proof of the existence of certain subtypes of this disease, such as the limbic‐predominant (LP) type. The hallmark of the LP type is a substantial number of neurofibrillary tangles found in the hippocampus and limbic areas, rather than the usual atrophy of the cortex (Murray et al. 2011).

These differences in brain changes relate to the various clinical features that include early‐onset episodic memory loss in the left posterior LP form, while in AD, there are broader cognitive changes. These conditions have common biomarkers, such as beta‐amyloid; however, the distribution and levels of such markers are different, making it difficult to diagnose the two forms (Tondo et al. 2021). In LP condition, patients show high TDP‐43 inclusion and unique tau protein distribution, which is indicative of the unique pathophysiology (Carlos et al. 2023). It should be noted that the presence of TDP‐43 inclusions causes increased hippocampal atrophy, which might explain the fast onset of LP in some patients.

Various neuroimaging differences also help to differentiate between the two forms, and LP patients have unique findings on radiographic imaging. These include severe atrophy in the medial temporal lobes and metabolic changes evident in FDG‐PET imaging. The use of imaging techniques, including tau‐PET, is useful in diagnosing and differentiating these two conditions.

One reason behind the continued discussion around LP's diagnostic category lies in conflicting views among researchers. A few see LP as its own disease, whereas some place it under the broader AD umbrella instead. How experts label it affects real‐world decisions in treating individuals. Errors happen often—postmortem data reveal numerous cases labeled as AD during life were later found to have LP changes (Butler Pagnotti et al. 2023). This points to a gap in current diagnostics—tools must adapt to capture finer clinical details, reducing misdirected treatments. Subtle shifts mark how cognitive decline unfolds in LP cases. Mini‐Mental State Examination (MMSE) results, for instance, tend to diverge in LP individuals, suggesting existing benchmarks lack precision (Hiya et al. 2024). Tracking changes over time shows LP‐related decline often advances at a reduced pace versus standard AD patterns. Speed of deterioration still depends heavily on accompanying issues such as vascular pathology.

An important conceptual distinction underlies this review. Neuropathologically, LP‐AD is defined postmortem by a disproportionate burden of neurofibrillary tangles in limbic regions relative to the neocortex, with preserved fulfillment of AD diagnostic criteria (Murray et al. 2011; Whitwell et al. 2012). In clinical practice, however, patients presenting with an LP amnestic syndrome are identified through clinical and radiological criteria, and such presentations may reflect LP‐AD, LP age‐related TDP‐43 encephalopathy (LATE‐NC), mixed pathologies, or other conditions (Corriveau‐Lecavalier et al. 2024; Butler Pagnotti et al. 2023). The studies included in this review largely identified patients based on these clinical, radiological, and histological criteria rather than exclusive neuropathological confirmation. The term LP is therefore used throughout to refer to this observable clinical and radiological presentation rather than to a specific postmortem confirmed subtype.

**FIGURE 1:**
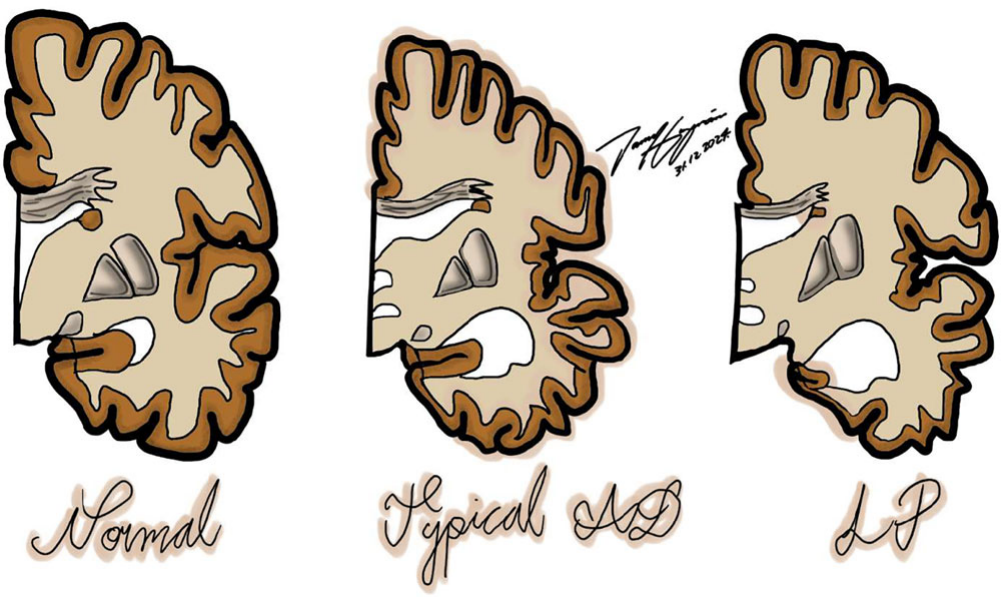
Comparisons between normal brain, typical Alzheimer's disease (AD), and limbic‐predominant (LP) variants. Typical AD is characterized by generalized brain atrophy, including the hippocampus, medial temporal lobe, and extensive cortical areas. In contrast, LPs show focal atrophy in limbic regions, such as the hippocampus and parahippocampal gyrus.

Even with advances in knowledge of neurodegenerative conditions, gaps remain in how we grasp initial symptoms and progression routes in logopenic primary progressive aphasia versus common Alzheimer's dementia (Figure 1). As such, this review works through findings from selected research to outline signs, brain imaging results, and tissue‐level markers separating logopenic cases from usual Alzheimer's forms. Differences seen across reports are then weighed carefully—not only to highlight distinctions but also to reflect on broader diagnostic groupings, given ongoing uncertainty around whether logopenic syndrome should align strictly with its underlying pathology labeled LP‐AD.

## 2 Methods

### 2.1 Search Strategy

**FIGURE 2:**
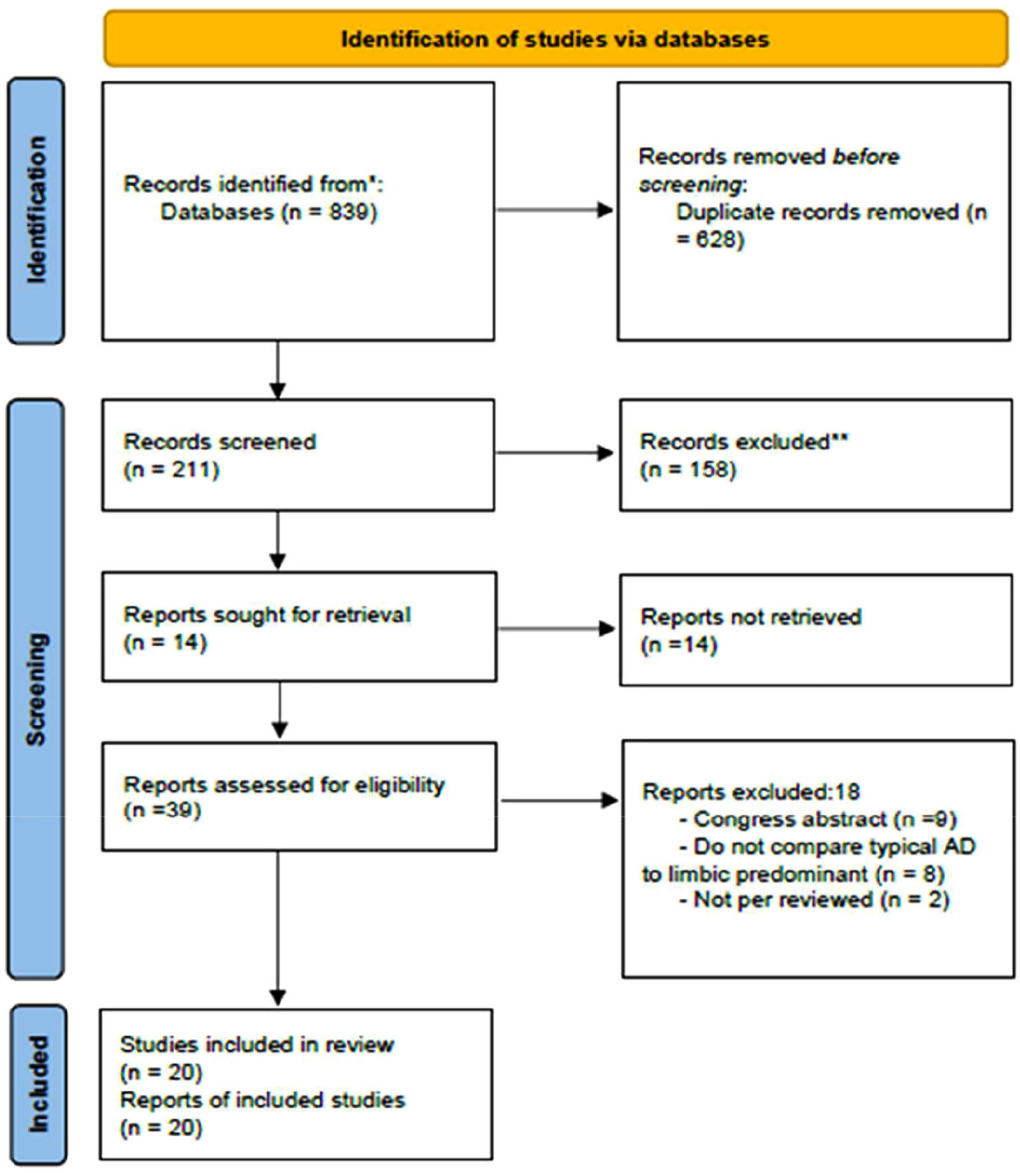
Flow diagram of study screening and selection.

This systematic review was conducted according to the Preferred Reporting Items for Systematic Reviews and Meta‐Analysis (PRISMA) guidelines (Liberati et al. 2009). The search strategy was conducted via Medical Subject Headings (MeSHs), including notable databases such as Medline, Embase, and Web of Science. MeSH terms were carefully aligned to find an intersection of LP and typical AD characteristics. More details can be found in Figure 2. Furthermore, this review was registered with the international Prospective Register of Systematic Reviews (reference number: CRD42024622149).

### 2.2 Study Selection

The study selection and screening process was conducted by three independent authors using Rayyan.ai. Researchers identified studies with reliable information about baseline characteristics; clinical, radiological, and immunohistological features and follow‐up times. The primary inclusion criteria for articles were (1) studies involving patients diagnosed with AD, specifically those classified into subtypes such as LP, typical, and hippocampal‐sparing subtypes on the basis of neurofibrillary tangle distribution and hippocampal atrophy; (2) studies that compared clinical and neuropathological features between different AD subtypes, particularly those that focused on neurofibrillary tangle density and distribution and hippocampal atrophy; (3) studies reporting on clinical presentation, age at onset, disease duration, cognitive decline, and neuropathological findings such as tau and TDP‐43 pathology; and (4) retrospective and prospective cohort studies, case–control studies, and neuropathological studies that provide data on the specified outcomes. Disagreements were resolved through discussion with a fourth reviewer.

### 2.3 Data Extraction

The data were collected by six authors via a predefined spreadsheet. The following items were extracted: authors, year of publication, journal, country/region, study design, sample size, age at diagnosis, sex, education in years, clinical presentation, duration of disease, imaging/histological features, biomarkers found, neurological measures used and their outcomes, follow‐up time, findings, and conclusions. Information on the included studies is provided in Table 1.

**TABLE 1:** Included studies.

Author‐year	Country/region	Study design
Tondo et al. (2021)	Italy	Retrospective cohort
Kouri et al. (2024)	USA	Retrospective cohort
Butler Pagnotti et al. (2023)	USA	Retrospective cohort
Hiya et al. (2024)	USA	Retrospective cohort
Lagarde et al. (2024)	France	Retrospective cohort
Carlos et al. (2023)	USA	Retrospective cohort
Levin et al. (2021)	Spain	Retrospective cohort
Nelson (2021)	USA	Retrospective cohort
Besser et al. (2020)	USA	Observational (cross‐sectional analysis using autopsy samples)
Tazwar et al. (2022)	USA	Observational study (cross‐sectional)
Gauthreaux et al. (2022)	USA	Retrospective autopsy cohort study
Mikhailenko et al. (2024)	Finland	Population‐based cohort study
Nag et al. (2020)	USA	Observational
Uretsky et al. (2021)	USA	Observational
Persson et al. (2017)	Norway and Sweden	Observational study
Mohanty et al. (2022)	Sweden, USA, Germany, Spain, UK	Observational study
Estades Ayuso et al. (2023)	USA	Observational study
Agrawal et al. (2021)	USA	Observational study
Machado et al. (2020)	Sweden, USA, Germany, Canada	Observational longitudinal study
Robinson et al. (2021)	USA	Retrospective cohort study

### 2.4 Quality Assessment

Two independent authors assessed the risk of bias and study quality via the Newcastle‐Ottawa Scale. Studies were evaluated for patient selection, comparability features, and exposure characteristics, with the results being within the range of 0–9, where 9 represents the highest quality value. More information can be found in Table 2.

**TABLE 2:** Results of the quality assessment via the Newcastle‐Ottawa Scale (NOS).

Studies	Items								Score
	Selection				Comparability	Exposure			
	1	2	3	4	1	1	2	3	
6		*	*	*	**	*	*	*	8/9
8	*		*	*	*	*	*	*	7/9
9	*	*	*	*	*	*	*	*	8/9
10	*	*	*		*	*	*	*	7/9
11	*	*	*	*	*	*	*	*	8/9
12	*	*	*	*	*	*	*	*	8/9
13	*	*	*	*	**	*	*	*	9/9
20	*	*				*	*		4/9
21	*	*	*	*	**	*	*	*	9/9
24	*	*	*		**	*	*	*	8/9
25	*	*	*	*	**	*	*	*	9/9
26		*	*	*	*	*	*	*	7/9
27		*	*	*	**		*	*	7/9
28		*	*	*	**	*	*	*	8/9
31	*			*	*	*	*	*	6/9
32	*			*	**	*	*	*	7/9
35		*	*		*	*	*	*	6/9
36	*	*	*	*	**	*	*	*	9/9
37		*	*	*	*	*	*	*	7/9
39	*	*	*	*	**	*	*	*	9/9

## 3 Results

### 3.1 Patient Information

After the initial review of 211 articles, screening was conducted, resulting in 21 articles, totaling 11,315 patients. Among these patients, 1178 (15.7%) presented with LP and 4159 (36.7%) with AD. A total of 5378 (47.6%) had a different presentation, including hippocampal sparing only and the association of LP and typical AD. The weighted average for education in years was 24.31 for LP patients and 17.15 for typical AD patients. The weighted average for age at onset was 72.33 for typical AD patients and 77.36 for LP patients. For the duration of disease, the weighted average for typical AD was 8.95, and it was 8.43 for LP. Additionally, there were no differences in clinical presentation, with cognitive impairment and memory deficits being the most commonly cited manifestations. More information can be found in Tables 3 and 4.

**TABLE 3:** Typical features of AD patients.

Study	Number of patients (n)	Female (%)	Age at onset (mean)	Education in years (mean)	Disease duration in years (mean)	Clinical presentation
Tondo et al. (2021)	62	56.4	71.14	13.95	4.05	N/A
Kouri et al. (2024)	1090	39.7	71	15.3	9.3	‐ AS ‐ MCI
Butler Pagnotti et al. (2023)	391	50.4	72.5	15.5	7.5	‐ MS ‐ BS ‐ AS
Hiya et al. (2024)	243	44.7	N/A	16.0 ± 0.2	N/A	‐ CI
Lagarde et al. (2024)	23	47.8	70.4	N/A	N/A	‐ AS ‐ CI
Carlos et al. (2023)	196	64	77	14	11	‐ CI
Levin et al. (2021)	87	38	73.2	15.5	N/A	‐ CI
Mikhailenko et al. (2024)	33	82.7	86.8	N/A	4.4	‐ CI
Uretsky et al. (2021)	213	74	81.3	80.7	16.2	‐ AS ‐ CI
Persson et al. (2017)	59	56	71.4	11.9	3.2	‐ CI ‐ AS
Mohanty et al. (2022)	31	25.8	80	16.1	N/A	‐ CI ‐ AS
Machado et al. (2020)	90	47.7	74.2	15.2	N/A	N/A
Robinson et al. (2021)	522	53.4	69.5	N/A	9.5	‐ CI ‐ AS

Abbreviations: AS, amnestic syndrome; BS, behavior symptoms; CI, cognitive impairment; MCI, mild cognitive impairment; MS, motor symptoms.

**TABLE 4:** Limbic‐predominant patient features.

Study	Number of patients (n)	Female (%)	Age at onset (mean)	Education in years (mean)	Disease duration in years (mean)	Clinical presentation
Tondo et al. (2021)	80	40	74.2	13.69	4.05	N/A
Kouri et al. (2024)	204	9.4	78.3^a^	14.2^a^	8.76^a^	‐ AS ‐ CI
Butler Pagnotti et al. (2023)	27	54.8	78.8	16.8	8.9	‐ MS ‐ BS ‐ AS
Hiya et al. (2024)	31	51.7	N/A	16.9	N/A	‐ CI
Lagarde et al. (2024)	17	35.3	77.3	N/A	N/A	‐ AS ‐ CI
Carlos et al. (2023)	167	59	75	15	9	‐ CI
Levin et al. (2021)	80	49	75.4	15.4	N/A	‐ CI
Nelson (2021)	34	N/A	N/A	N/A	N/A	‐ CI
Tazwar et al. (2022)	419	72.	N/A	15.7	N/A	‐ AS
Gauthreaux et al. (2022)	221	51.6	86.9	N/A	N/A	‐ CI
Mikhailenko et al. (2024)	20	75.9	87.3	N/A	2.5	‐ CI
Nag et al. (2020)	228	76.3	82.8	14.7	N/A	‐ CI ‐ AS
Uretsky et al. (2021)	57	67	83	16.3	N/A	‐ CI ‐ AS
Persson et al. (2017)	29	58.6	72.2	11.4	2.9	‐ CI ‐ AS
Agrawal et al. (2021)	1,670	68.62	N/A	16.9	N/A	‐ CI ‐ AS
Machado et al. (2020)	18	56	74.5	15.1	N/A	‐ CI ‐ AS

Abbreviations: AS, amnestic syndrome; BS, behavior symptoms; CI, cognitive impairment; MCI, mild cognitive impairment; MS, motor symptoms.

^a^ Weighted average of the mean values of three cohorts.

### 3.2 Imaging, Histological, Cognitive, and Biomarker Outcomes

Magnetic resonance imaging (MRI) and fluorodeoxyglucose positron emission tomography (FDG‐PET) are the most commonly used imaging techniques. Studies have shown greater hippocampal atrophy in LP patients than in typical AD patients. Additionally, lower levels of the amygdala and the entorhinal lobe were observed in LP patients compared with controls. In addition, typical AD patients presented diffuse cortical atrophy, whereas LP patients presented a tendency toward medial temporal lobe atrophy. LP patients presented higher rates of hypometabolism than typical AD patients did. T1‐ and T2‐weighted sequences were the primary structural MRI modalities used to assess morphological features such as hippocampal and cortical atrophy. Among the included studies reporting structural neuroimaging, MRI field strength ranged from 1.5T to 3T, which primarily affects image resolution and signal‐to‐noise ratio rather than the type of information captured. Lagarde et al. (2024) used a standardized 3T Siemens PRISMA system with a 3D T1‐weighted MPRAGE sequence at 1 × 1 × 1 mm^3^ voxel resolution, while Levin et al. (2021) and Machado et al. (2020) utilized Alzheimer's Disease Neuroimaging Initiative (ADNI)‐derived data acquired using a standardized T1‐weighted MPRAGE protocol across multiple 1.5T and 3T sites, with the resulting morphological volumetric features subsequently harmonized to account for residual site‐related variability. Persson et al. (2017) acquired MRI across 1.5T and 3T systems using either 3DT1 or T2 sequences depending on site availability, while Tazwar et al. (2022) employed MRI R2 relaxometry, a quantitative contrast that reflects tissue biological properties such as iron content rather than morphological or volumetric information, distinct in kind from the T1‐ and T2‐weighted structural sequences used in the other included studies. This variability in modality, field strength, and acquisition protocol across the included studies limits direct cross‐study comparison of quantitative imaging metrics. The specific metrics used to quantify hippocampal atrophy also varied substantially across studies. Volumetric approaches included automated FreeSurfer parcellation normalized to intracranial volume (Lagarde et al. 2024; Kouri et al. 2024), SPM8/VBM8‐based extraction using Harvard‐Oxford atlas‐defined hippocampal regions (Levin et al. 2021), and SPM anatomy toolbox hippocampal ROI masks (Machado et al. 2020). In contrast, Persson et al. (2017) and Machado et al. (2020) classified AD subtypes using the Scheltens medial temporal atrophy visual rating scale (scored 0–4) rather than volumetric segmentation. These methodological differences introduce systematic variability that precludes direct quantitative comparison of hippocampal measurements across studies and should be considered when interpreting the imaging findings presented in this review.

Additionally, the analysis of histological features revealed no differences in brain weight or in the presence of cerebrovascular diseases (e.g., arteriosclerosis) between the two groups, but LP patients showed a higher corticolimbic index. Biomarker composition across the studies revealed no large differences in the presence of proteins such as alpha‐ and beta‐amyloid, Lewy body, or neurofibrillary tangles, except for TDP‐43 inclusions, which were more associated with LP patients than with typical AD patients. Therefore, the distribution of neurofibrillary tangles was more restricted to the hippocampus, whereas in typical AD patients, they appeared more diffuse.

Finally, the most commonly used neurocognitive tests were the MMSE and the Clinical Dementia Rating, and no large differences were detected between LP and typical AD patients on either test (mean values of 20.18 for typical AD patients and 20.29 for LP patients). Therefore, the analysis of all other measures revealed that LP patients tended to perform better than typical AD patients, except for the trial‐making tests A and B. More information about those topics can be found in Tables 5 and 6.

**TABLE 5:** Imaging, histological, biomarker, and cognitive features of typical AD patients.

Study	Imaging findings	Histological findings	Biomarkers	Neurological measure used	Neurological measure scores
Tondo et al. (2021)	N/A	N/A	N/A	MMSE CDR global score	‐ MMSE 25.40 ± 1.81 ‐ CDR global score 0.50 ± 0.00
Kouri et al. (2024)	‐ Hippocampal volume: 1.7 (−2.3, −1.0) ‐ Tau‐PET parietal SUVr 1.3 (1.2, 1.4) ‐ Tau‐PET posterior cingulate and cuneus SUVr 1.4 (1.2, 1.4)	‐ Corticolimbic index 20 (19, 21) ‐ Brain weight, g 980 (910, 1125) ‐ Kalaria cerebrovascular disease scale 4.0 (2.0, 5.0)	‐ Thal amyloid ‐ Lewy body ‐ TDP‐43 positive ‐ Braak tangle	MMSE	‐ MMSE final score 13 (7, 19) ‐ MMSE longitudinal decline −1.4 (−3.4 to 0.2)
Butler Pagnotti et al. (2023)	N/A	‐ Microinfarcts ‐ Arteriosclerosis ‐ Hippocampal sclerosis	‐ Thal amyloid ‐ Lewy body	MMSE; LM1; LM2; BNT; Anim; Veg; DSF; DSB; DSym; TMT‐A; TMT‐B	Typical AD patients had worse scores in all measures except TMT‐A and TMT‐B
Hiya et al. (2024)	N/A	N/A	N/A	CDR; MMSE; LMI; LMD; DSF; DSB; TMT‐A; TMT‐B; WAIS; DS; BNT; Anim; Veg	Stepwise deleterious effect in all cognitive measures according to progressive levels
Lagarde et al. (2024)	Different atrophy levels in hippocampus, amygdala, entorhinal cortex, lateral and medial parietal cortex, lateral and medial temporal cortex	N/A	Low amyloid‐β42 levels and high tau levels	MMSE; CDR; Mattis DRS	Low scores in all measures, but similar to LATE‐NC patients
Carlos et al. (2023)	N/A	Hippocampal sclerosis; arteriosclerosis; infarcts; microinfarcts	Lewy body; neurofibrillary tangle; alfa and beta amyloid	MMSE; CDR‐SOB; UPDRS; TMT‐A and B; AVLT; WAIS‐BD; COWAT; NPI	‐ MMSE: 16 ‐ CDR‐SOB: 12 ‐ TMT‐A: 75 ‐ TMT‐B: 248 ‐ AVLT: 0 ‐ WAIS‐BD: 9 ‐ BNT: 34 ‐ COWAT: 23 ‐ NPI: 4
Levin et al. (2021)	Hypometabolism rate of 22.6% in FDG‐PET	N/A	AV45; CSF Aβ; CSF t‐tau	MMSE	MMSE: 23.2 (2.2)
Mikhailenko et al. (2024)	Relation between ADNC and Braak/CERAD scores	Braak stages V–VI; CERAD frequent scores	N/A	MMSE	MMSE scores significantly lower with ADNC and LATE‐NC overlap
Uretsky et al. (2021)	Differential neurofibrillary tangles burdens, but lower in hippocampus compared to limbic‐predominant	N/A	NFTs in hippocampal and cortical regions	MMSE	AD patients had worse scores and declined faster compared to limbic patients
Persson et al. (2017)	Different atrophy rates in temporal and frontal lobes	N/A	Amyloid β; phosphorylated tau	MMSE; CDR	‐ MMSE mean = 22.1 ± 4.3 ‐ CDR progression = 2.2 ± 2.0 annually.
Mohanty et al. (2022)	N/A	Different cortex atrophy levels	β‐Amyloid, tau, α‐synuclein, TDP‐43	MMSE; CDR; ADNI‐MEM	‐ MMSE: 18.16 ± 6.74 ‐ CDR: 1.339 ± 0.723 ‐ ADNI‐MEM: −1.27 ± 1.00
Machado et al. (2020)	Differential atrophy rates in basal forebrain, hippocampus, and precuneus	N/A	β‐Amyloid	MMSE	MMSE: 23.4
Robinson et al. (2021)	N/A	N/A	Amyloid‐β, tau protein	MMSE; CDR	Worsening in CDR was related to levels of cerebral amyloid angiopathy and ADNC

**TABLE 6:** Imaging, histological, biomarker, and cognitive features of limbic‐predominant patients.

Study	Imaging findings	Histological findings	Biomarkers	Neurological measure used	Neurological measure scores
Tondo et al. (2021)	N/A	N/A	N/A	MMSE; CDR	‐ MMSE: 25.73 ± 2.06 ‐ CDR: 0.50 ± 0.00
Kouri et al. (2024)	‐ Hippocampal volume −2.2 (−2.5, −1.6)	‐ Cortico Limbic Index 33 (32, 35) ‐ Brain weight, g 1100 (1000, 1130) ‐ Kalaria cerebrovascular disease scale 4.0 (2.0, 5.0)	‐ Thal amyloid phase 5 (5, 5) ‐ Lewy body disease 44/172 (26%) ‐ TDP‐43 positive (%) 38/56 (68%) ‐ Braak tangle stage VI (V, VI)	MMSE	‐ MMSE final score: 18 (8, 21) ‐ MMSE longitudinal decline −1.0 (−1.5, −0.23)
Butler Pagnotti et al. (2023)	N/A	Infarcts and lacunes, arteriosclerosis, microinfarcts, Lewy body pathology, hippocampal sclerosis, cerebral amyloid angiophaty	N/A	MMSE; LM1 and 2; BNT; Anim; Veg; DSF; DSB; DSym; TMT‐A and B;	Better scores in all measures, except TMT‐1 and 2, compared to typical AD patients
Hiya et al. (2024)	N/A	N/A	N/A	CDR; MMSE; LMI; LMD; DSF; DSB; TMT‐A; TMT‐B; WAIS DS; Anim; Veg; BNT	‐ Deleterious effects on global cognition and some aspects of memory, executive function, and processing speed
Lagarde et al. (2024)	‐ Reduced hippocampal, amygdala, and entorhinal volume compared to control ‐ Reduced hippocampal volume compared to typical AD	N/A	‐ Negative tau PET scans ‐ 3/17 limbic patients had slightly positive amyloid PET	MMSE; CDR; Mattis DRS	Worst scores in all domains, similar to AD
Carlos et al. (2023)	N/A	Hippocampal sclerosis; arteriosclerosis; infarcts; microinfarcts	‐ Lewy body ‐ Neurofibrillary tangle ‐ Alfa and beta amyloid	MMSE; CDR‐SOB; UPDRS; TMT‐A and B; AVLT; WAIS‐BD; COWAT; NPI	‐MMSE: 18 ‐CDR‐SOB: 8 ‐TMT‐A: 60 ‐TMT‐B: 240 ‐AVLT: 0 ‐WAIS‐BD: 12 ‐BNT: 41 ‐COWAT: 23 ‐NPI: 4
Levin et al. (2021)	Hypometabolism in 49.8%	N/A	AV45; CSF Aβ; CSF t‐tau; WMH	MMSE	MMSE: 23.4 (1.9)
Nelson (2021)	N/A	Hippocampal sclerosis	N/A	MMSE	MMSE: 17.7
Tazwar et al. (2022)	Lower R2 relaxation rate in the amygdala, hippocampus, and temporal lobe white matter, compared to AD patients	N/A	‐ TDP‐43 inclusions in the amygdala, entorhinal cortex, hippocampus, and neocortex	MMSE	MMSE: 19.4 ± 9.7
Mikhailenko et al. (2024)	Arteriolosclerosis in amygdala and hippocampus	N/A	TDP‐43 inclusions in amygdala, hippocampus, and frontal cortex	MMSE	MMSE: 18
Nag et al. (2020)	N/A	N/A	TDP‐43	MMSE	Similar outcomes to typical AD patients
Uretsky et al. (2021)	N/A	N/A	Higher hippocampal NFT burden compared to typical AD	MMSE	Similar outcomes to typical AD
Persson et al. (2017)	Medial temporal atrophy	N/A	Amyloid β, phosphorylated tau	MMSE; CDR	‐ MMSE: 22.1 (4.7) ‐ CDR: 4.9 (1.8)
Agrawal et al. (2021)	N/A	N/A	TDP‐43 in limbic regions	GCS; EM; SM; WM; PM; VA; PS	‐ GCS: − 1.00 (1.20) ‐ EM: − 0.93 (1.40) ‐ SM: − 1.28 (1.68) ‐ WM: − 0.75 (1.13) ‐ PS: − 0.75 (1.13) ‐ VA: − 0.57 (1.07)
Machado et al. (2020)	‐ Medial temporal lobe atrophy ‐ Basal forebrain degeneration	Hippocampal atrophy	‐ High NFT counts in hippocampus ‐ Amyloid‐beta	MMSE	N/A

MMSE: Mini Mental State Examination; CDR: Clinical Dementia Rating; LM1: Logical Memory 1; LM2: Logical Memory 2; BNT: Boston Naming Test; Anim: Animal Naming; Veg: Vegetable Naming; DSF: Digit Span Forward; DSB: Digit Span Backward; DSym Digit Symbol Substitution; TMT‐A: Trial Making Test A; TMT‐B Trial Making Test B; WAIS: Wechsler Adult Intelligence Scale Digit Symbol Substitution Test; DRS: Dementia Rating Scale; UPDRS: Unified Parkinson's Disease Rating Scale; AVLT: Rey Auditory Verbal Learning Test; COWAT: Controlled Oral Word Association Test; NPI: Neuropsychiatric Inventory; CSF: Cerebrospinal Fluid; WMH: White Matter Hyperintensities; ADNI‐MEM: Alzheimer's Disease Neuroimaging Initiative Memory score; ADNI‐EF: Alzheimer's Disease Neuroimaging Initiative Executive function; ADNC: Alzheimer's Disease Neuropathologic Changes; CERAD: Consortium to Establish a Registry for Alzheimer's Disease; CGS: Global Cognitive Score; EM: Episodic memory; SM: Semantic memory; WM: Working memory; PM: Perceptual memory; VA: Visuospatial ability; PS: Perceptual speed; NFT: Neurofibrillary tangles.

Abbreviations: ADNC, Alzheimer's disease neuropathologic changes; ADNI‐EF, Alzheimer's Disease Neuroimaging Initiative executive function score; ADNI‐MEM, Alzheimer's Disease Neuroimaging Initiative memory score; Anim, animal naming; AVLT, Rey Auditory Verbal Learning Test; BNT, Boston Naming Test; CDR, Clinical Dementia Rating; CERAD, Consortium to Establish a Registry for Alzheimer's Disease; COWAT, Controlled Oral Word Association Test; CSF, cerebrospinal fluid; DRS, Dementia Rating Scale; DSB, Digit Span Backward; DSF, Digit Span Forward; DSym, Digit Symbol Substitution; LM1, Logical Memory I; LM2, Logical Memory II; MCI, mild cognitive impairment; MMSE, Mini‐Mental State Examination; NPI, Neuropsychiatric Inventory; TMT‐A, Trail Making Test A; TMT‐B, Trail Making Test B; UPDRS, Unified Parkinson's Disease Rating Scale; Veg, vegetable naming; WAIS, Wechsler Adult Intelligence Scale Digit Symbol Substitution Test.

## 4 Discussion

This research analyzed 21 articles to characterize the clinical, radiological, and immunohistological features that distinguish the LP presentation of dementia from typical AD, as defined and reported across the included studies. Among all 11,315 patients, 15.7% presented with LP and 36.7% presented with typical AD. LP patients had a greater age at onset and higher education, but a similar disease duration. The clinical presentation was shown to be equivalent. Neuroimaging revealed greater hippocampus, amygdala, and entorhinal lobe involvement, in addition to greater hypometabolism in LP patients, whereas typical AD patients presented diffuse cortical atrophy. Histological analysis of biomarkers revealed a greater number of TDP‐43 inclusions and a greater hippocampal concentration of neurofibrillary tangles. Finally, in the cognitive domain, LP patients had a higher overall score than typical AD patients. These findings indicate important differences between the two presentations in neuroimaging, histology, biomarkers, and cognition, despite similar clinical features.

Before discussing individual findings, it is important to acknowledge a key interpretive challenge inherent to this literature. LP AD, as defined by seminal neuropathological work, is a postmortem entity characterized by disproportionate neurofibrillary tangle burden in limbic structures, with relative neocortical sparing, and fulfills full AD criteria (Murray et al. 2011; Whitwell et al. 2012). In clinical practice, however, an LP amnestic presentation can reflect multiple underlying pathologies, including LP‐AD, LP age‐related TDP‐43 encephalopathy (LATE‐NC), hippocampal sclerosis, or mixed pathologies. The studies included in this review employed varying diagnostic criteria, and a minority relied solely on postmortem neuropathological confirmation. As a result, the LP group in this review represents a clinically and pathologically heterogeneous population, and the term LP is used descriptively throughout. This heterogeneity is an inherent limitation of the available literature and is addressed further in the limitations section.

Age of onset, as reported in this review (mean 72.33 vs. 77.36), was one of the most important differences between the two groups. Two of the included studies reported statistically relevant data, indicating that this may be one of the main distinctions between typical AD patients and LP patients (Tondo et al. 2021; Butler Pagnotti et al. 2023). This information was also supported by several studies showing that LP patients had a later time of onset for dementia symptoms than typical AD patients did (Lagarde et al. 2024; Levin et al. 2021; Uretsky et al. 2021).

In terms of disease duration, we found no big differences between groups. In this context, the current literature supports this finding, as many studies have shown that LP and typical AD patients tend to have similar disease duration (Tondo et al. 2021; Butler Pagnotti et al. 2023; Uretsky et al. 2021), except for Carlos and collaborators, who reported a difference in typical AD plus TDP‐43 encephalopathy patients (*p* = 0.02) (Agrawal et al. 2021).

Moreover, education was also an important factor for differences between the groups in this systematic review. However, this finding may be related to the weighted average performed in this study, as many studies have not shown large differences (Tondo et al. 2021; Butler Pagnotti et al. 2023; Lagarde et al. 2024; Levin et al. 2021; Carlos et al. 2023), and one could not relate education to LP stages (Nag et al. 2020).

Furthermore, this study revealed many differences between LP and typical AD patients, especially analyses focused on the hippocampus, amygdala, and medial temporal lobe (Whitwell et al. 2012). In this context, studies have also revealed a correlation between those differences and other brain regions, showing that higher rates of basal forebrain atrophy are more common in LP individuals than in those with typical AD (Lagarde et al. 2024). However, similarities were also found: a retrospective cohort revealed a reduction in the volume of the nucleus basalis of Meynert, a known early marker of typical AD, and different stages of atrophy in the hippocampus, amygdala, and entorhinal lobe in both LP and typical AD patients (Robinson et al. 2021). Additionally, studies have shown that overall hypometabolic states are greater in LPs but, when present in typical AD patients, are more strongly associated with progression to dementia (Tondo et al. 2021). Nevertheless, a recent cohort showed that LATE‐NC patients tended to have lower R2 relaxation rates on MRI, especially in advanced stages, in the temporal, frontal, and occipital lobes and basal ganglia, with the hippocampus, parahippocampal cortex, entorhinal cortex, and temporal lobes being the most affected (Agrawal et al. 2021). Finally, our findings suggesting greater hippocampal impairment were corroborated by two studies that reported that a greater hippocampus volume and a higher cortical concentration of flortaucipir PET SUVR were associated with less impairment of the limbic system and that hippocampal sclerosis was greater in LATE‐NC patients than in typical AD individuals (Lagarde et al. 2024; Kouri et al. 2024).

In addition, the histological features were highly diverse across the included studies, which is a limitation of this review, as these analyses could not be synthesized. However, many studies have identified important features, such as a tendency for LP to be associated with arteriosclerosis but less so with amyloid angiopathy (Woodworth et al. 2024). Additionally, although studies comparing AD and LATE‐NC using neurocognitive tests have demonstrated significant differences, with LATE‐NC showing a more circumscribed, memory‐predominant pattern and slower decline than the broader deficits of typical AD, these differences alone cannot reliably distinguish the two at the individual level

Moreover, concerning biomarkers, all the studies mentioned above revealed amyloid pathology in both typical AD and in its LP variant (Lagarde et al. 2024; Levin et al. 2021; Carlos et al. 2023; Machado et al. 2020; Persson et al. 2017). It was measured in vivo with amyloid‐PET imaging and postmortem with amyloid staining; therefore, the role of amyloid protein as one of the key biomarkers in the AD spectrum was proven (Kouri et al. 2024; Agrawal et al. 2021). Alongside the amyloid protein, the study shows that the tau protein was present in both variants as well, which was confirmed using tau‐PET imaging and postmortem examination of neurofibrillary tangles according to the Braak stage criteria. There are other proteins, such as alpha‐synuclein and Lewy bodies; however, the prevalence of their occurrence did not differ significantly among the subtypes (Kouri et al. 2024; Carlos et al. 2023; Mohanty et al. 2022). Another factor worth mentioning is TDP‐43 inclusions as the distinctive biomarker of the two subtypes. Even though it occurs in some cases of typical AD, it is mostly related to the LP variant as shown in various immunohistochemical studies (Kouri et al. 2024; Nag et al. 2020; Agrawal et al. 2021; Mikhailenko et al. 2024). They were mostly present in limbic brain areas such as hippocampus, amygdala, and entorhinal cortex, while, in case of the typical AD, they are diffusively present throughout the cortical areas. Also, these findings correlate well with the results of imaging tests, according to which LP showed signs of hippocampal atrophy and hypometabolism in the medial temporal lobe compared to diffusively affected cerebral cortexes of typical AD cases (Uretsky et al. 2021; Nag et al. 2020; Agrawal et al. 2021; Persson et al. 2017; Mohanty et al. 2022; Robinson et al. 2021). Recent advances in computational neuroimaging have substantially expanded the capacity to identify and characterize AD subtypes in vivo. Vogel et al. (2021) applied a machine learning subtype and stage inference algorithm to tau‐PET data from 1612 individuals and identified four distinct spatiotemporal trajectories of tau deposition, including a LP pattern and a medial temporal lobe‐sparing pattern that correspond closely to the presentations examined in this review. Complementing this, Levin et al. (2021) employed data‐driven hierarchical clustering of FDG‐PET data from biomarker‐confirmed AD patients.

Yet, several limitations should also be discussed. The differences in sample sizes, lack of longitudinal studies, and the existence of publication bias are among the main limitations of this study. Moreover, the inclusion criterion emphasized studies assessing clinical, radiological, and histopathological criteria across presentations instead of papers discussing exclusively autopsy‐verified pathology of LP‐AD, which was done because there was no adequate clinical literature regarding the issue under consideration. However, another limitation is associated with the need to conduct future studies involving advanced imaging techniques and standardized histological analysis to determine if LP is a stand‐alone condition or a specific form of AD. Moreover, the issue should be explored by researchers in relation to young people because their differentiation is critical from the point of view of early diagnosis.

## 5 Conclusion

Our analysis of clinical, radiological, and immunohistological features revealed significant differences between LP and typical AD presentations. However, those findings alone cannot reliably determine whether both presentations are stages of the same pathology or different diseases. More studies need to explore this field to further examine this topic.

## Author Contributions

**Guilherme Linha Secco**: conceptualization, investigation, writing – original draft. **Kashif Qureshi**: writing – review and editing, visualization, validation. **Murtaja Satea Shafeea**: writing – review and editing, methodology, conceptualization. **Pedro Henrique Karpinski**: validation. **Kim Wouters**: formal analysis. **Mateus Cordeiro Merlim da Silva**: software, data curation. **Sravan Reddy Ganta**: data curation, supervision. **Enzo von Quednow**: software, validation. **Edgar Daniel Guzmán‐Ríos**: validation, investigation. **Khalil St Brice**: investigation, formal analysis. **Mahi Dsouza**: visualization, formal analysis. **Ailton Fernandes Junior**: data curation, software. **Noor Najah Noori**: validation, methodology. **Bipin Chaurasia**: writing – review and editing, visualization, supervision. **Kivanc Yangi**: writing – review and editing, visualization, validation.

## Funding

The authors have nothing to report.

## Ethics Statement

The authors have nothing to report.

## Conflicts of Interest

The authors declare no conflicts of interest.

## Data Availability

Data sharing not applicable to this article as no datasets were generated or analyzed during the current study.
